# How well does Anorexia Nervosa fit with personal values? An exploratory study

**DOI:** 10.1186/s40337-016-0109-z

**Published:** 2016-07-19

**Authors:** Úna Mulkerrin, Bryony Bamford, Lucy Serpell

**Affiliations:** Research Department of Clinical, Educational and Health Psychology (Torrington Place Site), University College London, Gower Street, London, WC1E 6BT UK; Present address: Dublin Adult and Child Therapy Centre, 11 Sandyford Office Park, Blackthorn Avenue, Sandyford, Dublin 18 Republic of Ireland; The London Centre for Eating Disorders and Body Image, Hanover Square, London, W1S1HT UK; Eating Disorder Service, North East London NHS Foundation Trust (NELFT), Porters Avenue Health Centre, 234 Porters Ave, Dagenham, RM8 2EQ UK

**Keywords:** Anorexia nervosa, Personal values, Motivational tools, Enhancing motivation to change, Ambivalence

## Abstract

**Background:**

Despite an increasing clinical interest in the use of personal values as a motivational tool in psychological therapies for Anorexia Nervosa (AN), research is limited. This study explored personal values among individuals with AN, with a particular focus on the ‘fit’ between participants’ values and their AN.

**Methods:**

A qualitative research design was employed in this study. In-depth, semi-structured interviews were carried out among eight female outpatients and inpatients with a diagnosis of AN or Eating Disorder Not Otherwise Specified – AN type (EDNOS-AN type). Data was analysed using Interpretative Phenomenological Analysis (IPA; Smith, Jarman & Osborne, 1999).

**Results:**

Three super-ordinate themes emerged from analysis: ‘*Balancing Values’* (difficulty finding balance in relating to and acting on values)*, ‘Congruence and Clashes between AN and Values’* (experiences of AN representing a mixed-fit with values) and *‘From Ambivalence to Motivation’* (ambivalence toward both AN and recovery – in the context of its mixed-fit with values – and experiences of values as a motivational tool in recovery).

**Conclusions:**

Study findings support a role for psychological therapies in working with personal values as a means of promoting recovery in AN, through supporting individuals to explore AN’s workability in the context of their values. Further research investigating the optimal stage of treatment to work with values as a motivational tool is warranted.

## Background

Anorexia Nervosa (AN) is widely considered qualitatively different to other psychological disorders. This is attributed to an *active* engagement in the patterns that maintain the disorder and over-evaluation of AN’s symptoms [1]. Individuals with AN generally consider these highly valued symptoms to be consistent with their self-concept and central to their ‘ideal’ identity, hence the use of the term ‘ego-syntonic’ to describe this phenomenon [2]. For example, an individual with AN for whom discipline is a value might perceive themselves to achieve a greater sense of discipline through severe dietary restriction. When this individual achieves a significantly low weight through this behaviour, they may perceive this as tangible evidence that their AN symptoms bring them closer to a heightened sense of discipline. Indeed, it has been argued that some symptoms of AN are so fiercely ego-syntonic that “low weight and restrictive eating are not merely accepted as consistent with the ‘real self’, but valued as accomplishments of the ‘best self’” [3] (p181). Recent research suggests that individuals who exert extreme effort applying values or principles with a perceived high social value – a phenomenon referred to as *pliant valuing –* may be more vulnerable to developing AN [4, 5].

Through AN’s ego-syntonicity, its symptoms often become viewed by the individual as the solution to – rather than the cause of – emotional difficulties. This represents a paradox; individuals’ AN-related behaviours are intended to elicit a positive mental state, including self-confidence, control and emotional stability, through – for example – demonstrating the ability to adhere to strict, rigid rules relating to food, eating and weight [6, 7]^.^ However, these efforts tend to have the inadvertent effect of causing and prolonging suffering, risking physical health and increasing obsessionality (for example via set-shifting difficulties and/or perseveration) through being in a prolonged state of semi-starvation [8–10]. Semi-starvation effects are notoriously difficult to disentangle from symptoms of AN itself and the personality traits of those who develop it [11, 12]. However, it is evident that through striving to achieve AN-related goals – which are by nature difficult to achieve – a resultant tendency to compromise or neglect more over-arching personal values (including spiritual, interpersonal, moral and career-related values) becomes apparent. Previous qualitative research highlighted the detrimental effect AN symptoms have on social, health, interpersonal, emotional and psychological areas of functioning, despite perceived benefits of the disorder [6]. Self-perceived deficits in functioning have also been identified in intimate relationships, family relationships, health, general well-being and work and education [13]. Semi-starvation may play a role in these documented effects, considering it has produced similar physiological, psychological, emotional and social effects in healthy, ‘non-anorexic’ individuals, including conscientious objectors involved in a medical experiment [14] and those pursuing Caloric Restriction for Longevity [15].

The detrimental consequences of persistent symptoms of AN are confounded by a strong positive valuation on self-control and thinness that serve to maintain the disorder and can impair motivation to change [16–18]. Low motivation and readiness for change are associated with more severe eating disorder symptoms and greater internalising problems, including depression and anxiety [19]. Impaired motivation is also predictive of poorer clinical outcome in AN [20]. For this reason, it is important to focus on identifying ways of addressing feelings of ambivalence toward and enhancing motivation to actively engage in treatment and recovery. In this context, motivational interventions [21, 22] including Motivational Interviewing (MI) and Motivational Enhancement Therapy (MET) have been identified as promising approaches to treating AN [23, 24]. From a motivational perspective, Vitousek and colleagues argued that highlighting the dissonance between conflicting values among individuals with AN can enhance motivation for treatment and symptom change [1]. Their rationale for this is the restoration of congruence between ‘principles and practices’. This is seen as a starting point for challenging beliefs about the ‘rightness’ of AN, which can be built upon by encouraging individuals to work toward their values, thus helping to reduce AN behaviours and facilitate positive change. Two measures that have been developed specifically for use with individuals with AN – the Pros and Cons of Anorexia Nervosa Scale (P-CAN) and the Decisional Balance Scale for Anorexia Nervosa – which can be used to support individuals to identify their level of motivation to change, engage in motivational exercises aimed at exploring pros and cons of AN and consider negative aspects of AN that they might want to work to leave behind through full engagement in treatment [7, 25].

Acceptance and Commitment Therapy (ACT) [26] is another psychological therapy that uses personal values to enhance motivation to change. Among ACT’s six core principles is a focus on values; defined in ACT as the things in life that are meaningful and significant to the individual, with a view to facilitating a behavioural shift toward a way of living that is more in line with one’s own values [27]. The use of ACT is receiving increasing interest and attention as a treatment for eating disorders [28, 29]. There is some preliminary evidence that ACT is beneficial for treating AN as well as body image difficulties & sub-clinical eating concerns [30–34].

While highlighting dissonance between conflicting values has been theorised as being important to successful psychological interventions for AN [1], there is currently a dearth of research exploring experiences of this phenomenon amongst individuals with AN. Thus, the present study will qualitatively explore the experience of core personal values among individuals with AN and in particular, the perceived fit between AN and core values.

Specifically, this study will explore the way individuals with AN experience their core personal values and their understanding and experience of (1) the valued aspects (if any) of their eating disorder, (2) their non-weight related values, and (3) conflicts between opposing values and the fit between their eating disorder and personal values. For the purposes of this study, values were defined as things on which people place a strong sense of importance or meaning [35].

## Method

### Design

This study employed a qualitative methodology involving in-depth, semi-structured interviews with participants, using Interpretive Phenomenological Analysis (IPA) [36]. IPA has been described as “a systematic and practical approach to analysing phenomenological data”, which focuses on studying a person’s experience and subjective perceptions of a phenomenon using a ‘bottom-up’ approach that attempts to avoid prior assumptions [37] (p81). It was felt that this particular qualitative methodology would best suit this study due to the aim of exploring the phenomenon of personal values, and its fit with AN, from the perspective of individuals with AN.

### Participants

The eight adult female participants (six outpatients and two inpatients) were receiving treatment at two London-based Eating Disorder Services (EDSs). Four participants had a DSM IV diagnosis of Anorexia Nervosa (AN) Restricting Type, four of Eating Disorder Not Otherwise Specified – AN Type (EDNOS-AN type). Two of the EDNOS-AN type participants had recently migrated from a diagnosis of AN because their Body Mass Index had exceeded 17.5, hence their inclusion in the current study as individuals recovering from AN. All participants will be discussed in the context of AN from here on in. Table 1 (below) provides a summary of participants’ demographic information.

**Table 1:** Summary of participant demographics

Name	Age	Diagnosis	BMI	Service status	Therapy Status	Ethnicity	Years since ED onset
Sarah	23	AN	15.6	Outpatient	No	White British	11
Jean	32	AN	17.1	Outpatient	Yes	White British	6-7
Laura	29	EDNOS-AN type	18	Outpatient	Yes	White British	14
Sophie	40	AN	14.3	Outpatient	Yes	White British	5
Heather	31	EDNOS-AN type	18	Outpatient	No	White British	17
Emily	20	EDNOS-AN type	20	Outpatient	Yes	White British	2.5
April	21	EDNOS-AN type	18	Inpatient	Yes	White Irish	10
Shauna	30	AN	14.6	Inpatient	Yes	White British	22

Name = Pseudonym; AN = Anorexia Nervosa; EDNOS-AN type = Eating Disorder Not Otherwise Specified-Anorexia Nervosa type; BMI = Body Mass Index at time of research interview

Apart from one participant who was closely monitored by a specialist mental health nurse and other health professionals in her eating disorder service, all participants were engaged in some form of psychological therapy, but were at different stages in the therapeutic process.

Exclusion criteria were: abuse of a substance to the degree that it would meet the criteria for an addiction, florid psychotic symptoms, organic psychosis or severe learning disability.

Initially, the aim was to recruit individuals (male or female) who were (i) engaged with, and receiving regular monitoring from health professionals at their EDS, but who were not engaged in psychological therapy, or (ii) in their first four to six weeks of psychological therapy. It was important for participants to be actively engaged with services; from an ethical perspective, it was judged unwise to carry out in-depth, emotive interviews with individuals who were lacking access to a containing forum in which to discuss potentially difficult feelings emerging from the process. Participants in later stages of psychological therapy were initially excluded from the study to control for any potential impact of therapy on their values. However, due to recruitment difficulties, along with a finding that almost all potential participants had received at least one course of psychological therapy previously, it was decided that individuals at any stage of therapy could participate.

The recruitment procedure involved clinicians within the two services identifying individuals who met inclusion criteria for participation in the study. Eligible individuals were then invited to participate. Participants all provided written consent to be included in the research study and for their anonymised data to be published. Ethics approval for the study was granted by the NHS North London Research Ethics Committee.

### Semi-structured interview

Participants were informed that the interview would ask about their core personal values, and the way these relate to their eating disorder. As previously mentioned, values were defined as things on which people place a strong sense of importance or meaning [35]. Interviews lasted between one hour and one hour and 50 min. Interviews were recorded using a digital voice record. The interview comprised a phenomenological exploration of a number of areas, including:

- Participants’ core personal values,
- Participants’ understanding and experience of the valued aspects (if any) of their eating disorder,
- Participants’ understanding and experience of their non-weight related values, and
- Participants’ understanding and experience of conflicts between opposing values, and the fit between their eating disorder and personal values.

The above areas included more detailed prompts and questions in the actual interview schedule, whilst also allowing flexibility to be guided by the participants responses. Interview questions were piloted with three individuals who met study criteria. Feedback from this was incorporated into the final version of the interview schedule. The three individuals with whom the questions were piloted did not participate in the study.

### Data analysis

Data were analysed using Interpretive Phenomenological Analysis [36], which allowed the researchers to explore the phenomenon of personal values as they relate to AN, from a ‘bottom-up’ perspective, being led by the experiences of the participants.

Audio-recordings of interviews were transcribed verbatim. Any identifiable information was changed in order to ensure anonymity. Once transcribed, interviews were analysed to draw out and understand the meanings from the text. Initial notes were written on the text, using different colours to indicate descriptive, linguistic and conceptual comments. As suggested by Smith, Flowers and colleagues, these notes were then transformed into emergent themes, which were subsequently linked together and organised into clusters to generate higher order (super-ordinate) and lower order categories of themes [38]. The extracted themes were then compared across participants to create a list of super-ordinate themes, or ‘domains’, within which a number of lower order themes fit.

### Credibility checks

A number of processes – described here as ‘credibility checks’ – were put in place to increase the rigor of this qualitative study. These included ‘virtual auditing’ [36], which involved retaining all documents created during the data collection, analysis and reporting stages so that in theory (for example at a later date) an independent individual could follow every step of the study’s process in a meaningful and coherent way; ‘analytic auditing’ [36], which involved the two co-authors checking the results of the qualitative data analysis against the raw data; and ‘testimonial validity’ or ‘member checking’, which involved checking the initial emergent themes with participants and incorporating their written feedback into the final results [39]. This process enabled participants to have some involvement in the process of theme generation, ensuring that interpretations made are viable and serve as a reasonable representation of their shared experiences.

## Results

Three super-ordinate themes, or *domains* were identified. Within each *domain*, a number of *themes* and *sub-themes* emerged (see Table 2). Each domain will be presented followed by a description of its associated themes and sub-themes and supporting quotes from participants, using pseudonyms as identifiers. Three dots (…) will be used to indicate unrelated omitted text in quotes, and (p) will be used to indicate a significant pause. Underlined text indicates a word that the participant placed emphasised on.

**Table 2:** List of domains, themes and sub-themes

Domain	Theme	Sub-Theme
1. Balancing values	1.1. Extremes in values (8)^a^	
	1.2. Clarification of values (4)	
2. Congruence and clashes between AN and values	2.1. Perceived congruence	2.1.1. AN as a physical and behavioural representation of principles (8)
	2.2. Perceived incongruence	2.2.1. *“You can’t have both”* - AN as a saboteur of values (7)
		2.2.2. *“I’m that much in control that I’m out of control” –* Paradoxes in AN (8)
3. From ambivalence to motivation	3.1. Ambivalence toward AN (6)	
	3.2. Ambivalence toward recovery (7)	
	3.3. Values as a beacon of hope (5)	

^a^The prevalence (i.e. the number of participants for whom a particular theme emerged) is indicated in brackets to the right of each theme

### Domain 1: balancing values

In-depth analysis of participants’ transcripts revealed a number of difficulties relating to balance in the context of personal values; specifically, striking a balance in the way they relate to and pursue their personal values.

### Extremes in values

It was common for participants to describe upholding, or ‘living in line with’ their principles in an extreme manner. Participants described feeling compelled to act in a way that fits with principles they consider virtuous, to the detriment of other valued life areas. When Jean acts in line with the value she places on hard work and commitment, she comes to realise it is extreme, as compared to other people’s ‘normal’ behaviour:

*I’ve got to put my best into it, [I: Yeah] otherwise I’m not good enough…you can feel proud about what you’ve done because you’ve put your best into it.* (Jean)

Similarly, Shauna described an experience of acting in line with her values of productivity and achievement in the extreme, at the expense of freedom and relaxation:

*And the whole time I pushed myself and pushed myself to the max, and nothing is ever good enough in that job anyway but nothing is ever good enough for me.* (Shauna)

Sarah described an experience of feeling compelled to pursue her values in a perseverative way, to the extent that her basic health needs become compromised:

*I’m a perfectionist, so it can be quite stressful…I’ll just work as hard as I can to get the best I can. With my Art A-Level I got – I didn’t lose any marks…but then, I did eight sketch books and, you know, I had two walls of paintings and…sculpture and…I didn’t sleep for about (p) for a year, doing all that.* (Sarah)

Dichotomous thinking about values was observed, through Laura’s health-related values, which indicate that she can only feel she is living in line with her value on physical health if she is severely restricting, while eating is considered ‘unhealthy’:

*I exercise and I really restrict [I: OK] what I eat on healthy days…if someone says to me “we’re going out in three weeks” (l) I’ll be “oh my gosh, I’m going to have to be unhealthy”* (Laura)

### Clarification of values

Four participants identified experiencing a difficulty in identifying or clarifying their personal values. This appeared to be associated with distress and participants seemed to suggest that AN was a key factor in stifling their ability to clarify their overarching values:

*I’ve always felt like I’ve put on two masks [I: Right] – my eating disorder mask and the other side – and I just, I really don’t know, I’ve lost (l) what I value really [I: Mm] and what I’m interested in or, the things that used to make me happy or that I love.* (Shauna)

Laura described the way in which her eating disorder has made her question and doubt her passions and values in life, as she struggles to act in line with her values of creativity and hard work through her eating disorder:

*I get annoyed with myself because even, like, my great passion, I can’t even do it. I let food and thinness get in the way…and I think if I really liked it as much as I say I like it then I should be able to do it (p) but I don’t* (Laura)

This distressing experience of lack of clarity regarding values appears to be linked with participants’ stage in recovery. Those who described AN as interfering with their ability to identify and clarify values were at a stage where their eating disorder had a particularly powerful grip. Sarah’s description of this phenomenon pointed to her significantly low weight as a potential cause of this confusion, which may indicate that starvation effects play a role:

*You go into a kind of blur when you are at a low weight. It makes it hard to think coherently, which is a barrier to recovery…your brain is in chaos so you turn back to the anorexia to get clarity.* (Sarah, in comments on the themes)

For those who felt more confident in their ability to overcome AN, this phenomenon was not articulated during the interview. Heather, who has been recovering from AN for the past year, described how a strengthening ‘sense of self’ and an increased ability to clarify and act on her values has been a key element of her recovery. She talked about her increased ability to pursue the value she places on living a full life, which has occurred alongside progress in recovery:

*This is my third chance at getting better [I: Yeah], I have to be alive (p) I’m obviously here for a purpose, and realizing that (p) (being thin) didn’t change anything. It did at the time, but the things I’ve got now, I couldn’t do then…I mean there are a lot of times where, if those things hadn’t become more pronounced as I got better, I probably would (p) have been ill still.* (Heather).

Regarding the mixed experiences of participants in terms of their ability to clarify their values, this appears to be linked with stage in, or attitude toward recovery. Those who described difficulty with this appeared to experience AN as having a stronger grip on them. They also had lower Body Mass Indexes, suggesting that they may have been more cognitively affected by semi-starvation than those who were more able to clarify their values.

### Domain 2: congruence and clashes between AN and values

The results indicate that participants in this study experienced a mixed fit between AN and their personal values. For example, congruence was observed in the form of AN representing a physical and behavioural manifestation of some intrapersonal values, while on the other hand incongruence was observed in the form of AN sabotaging participant’s ability to live in line with some interpersonal values.

### Perceived congruence

Participants identified a number of ways in which AN and its associated behaviours and symptoms are experienced as congruent with their personal values. They described how physically (through extreme thinness) and behaviourally (through severe dietary restriction and restraint) AN is experienced as a manifestation, or symbol of some of their core values, including self-control, discipline, hard work, achievement and willpower.

#### AN as a physical and behavioural representation of principles

Participants described experiencing AN as a symbol of their commitment to pursuing their values, or as a vehicle through which these values are upheld. Emily described how being thin has been a representation of the commitment she shows to her value on working hard and achieving:

*I suppose I’m quite competitive as well so to be the thinnest person in the room is quite an achievement.* (Emily)

April described experiencing a sense of congruence between AN and some of her intrapersonal values, in that severe restriction acts as a physical manifestation of those virtues, or valued attributes:

*When I don’t eat and when I do those things it’s kind of like a buzz, in the sense that all the positive aspects that I think about myself – that (p) I have willpower, I’m strong, I’m independent, I feel attractive, I feel confident – when I do those things like that it feeds into those things.* (April)

All participants also described how AN fits with the value they place on control. While AN behaviours and symptoms are experienced as providing a sense of control, it is unclear whether the control they speak of is better described as a ‘true’ value, a coping mechanism, or a means of engaging in experiential avoidance. Striving for control in the context of AN seemed to be valued by some participants because it offers a way of coping with, or avoiding unwanted internal experiences (i.e. thoughts or feelings). It thus appears that control may have different meanings and functions across participants. Shauna described how AN provides a way for her to act in line with the value she places on control:

*Shauna: It’s just being in control – that’s another thing that I value (l)…I feel like I have to be in control of everything.*

*I: OK, and how well do you think the anorexia – the anorexia-related behaviour – fits with that value that you place on control?*

*Shauna: Very, very much, because eating disorders are kind of all about control*

However, for Heather, gaining control through AN seems to represent more of a coping mechanism than pursuing a value:

*So it was very lonely and I couldn’t tell (my parents) that, and then it just got to a point where… (p) hang on, I’m feeling out of control but I’m in control of something with the food.* (Heather)

### Perceived incongruence

Analysis also revealed a number of ways in which AN is perceived as incongruent with participants’ personal values. This became apparent in two contexts; (i) AN’s sabotaging effect on participants’ ability to act in line with their values and (ii) the paradoxical experience of AN seeming congruent with values, but in reality compromising them. Participants’ descriptions of the incongruence of AN with their values highlights the ego-dystonic aspects of AN. Most prominently, participants’ descriptions indicated that their interpersonal values, including having close friendships and family relationships, caring for others and having fun and socialising with others are most overtly compromised by AN. As well as an experienced incongruence between AN and interpersonal values, participants described a paradox, whereby AN is intended as a way of acting on some of their values, particularly control, but actually has the inadvertent effect of alienating them further from them. This incompatibility was often associated with distress; it appears that participants find it difficult to tolerate and reconcile the misfit between AN and these values (see also Theme 1.2: Tensions and Conflicts Between Values).

#### “You can’t have both” – AN as a saboteur of values

Participants described experiencing AN as a saboteur of their personal values, in the sense that it stops them from pursuing them as they would like to. Heather described the sheer incompatibility of AN and her values:

*You can’t have them both* (AN and her values of closeness to others and living a full life) *– you just can’t – because you’re too caught up with it – it takes away everything.* (Heather)

Emily, who described closeness in relationships as a strong value for her, also identified AN’s sabotaging effect on this value. This represents a major incongruence in the context of the importance she places on investing in family relationships and friendships:

*Um (p) the eating disorder sometimes takes precedence, especially lately…if there’s a social event that would require me skipping exercising for a day or, you know, I would turn down the offer of going for meals, so that kind of restricted my social life, and because I became a bit reclusive, I got caught up in my own head that going out to parties I’d be like “no, I don’t want to”.* (Emily)

#### “I’m that much in control that I’m out of control – paradoxes in AN

AN and its associated behaviours and symptoms presented a paradox of values. Discussion with participants highlighted how AN is intended to represent a way of acting on their values. However, it appears to have the paradoxical effect of compromising them.

*With control (through AN) there’s something of security, but I’m in control but out of control, if that makes sense? Because the control I’ve got has made me out of control in a way.* (Jean)

*You think you’re in control but as I was saying before you spiral out of control – so it’s not totally reliable and everything.* (Shauna)

Through conversation with Laura, it became apparent that she uses restriction as a way of allowing herself to feel able to act on her interpersonal values (in this case socialising with loved ones at Christmas). However, it was equally apparent that her strategy had the inadvertent effect of compromising her ability to socialise in the here-and-now:

*I just feel I can’t enjoy Christmas if I don’t do this (restriction) beforehand…I won’t go out – I won’t – and I won’t make any plans.* (Laura)

### Domain 3: from ambivalence to motivation

While participants in the study expressed ambivalent feelings toward both AN and recovery, there was a clear indication that exploring personal values could be a powerful tool in considering the workability and sustainability of AN and thus promoting motivation to change. Participants’ descriptions highlighted two internal battles: (i) the elements of AN that represent their values versus the elements of AN that prevent them from acting on their values and (ii) the elements of recovery that would make it easier to act on their values versus the difficulty letting go of the valued aspects of AN. This seems to fit with previous research investigating ambivalence and motivation in AN [40, 41]. However, it appears that the phenomena of ambivalence and motivation to change are not as straightforward as weighing in the balance the pros and cons of holding onto, versus letting go of AN. Seven participants in this study reported a desire to recover from their eating disorder, but this desire was confounded for some by their perceived powerlessness against AN.

### Ambivalence toward AN

Participants reported experiencing some elements of AN as highly valued, with other elements described as paradoxical, distressing and unwanted. This expression of mixed feelings in relation to AN and its perceived impact, costs and benefits indicated a clear sense of ambivalence among participants toward AN. The quotes below highlight the extent of the internal battle experienced by participants, who on the one hand seemed to perceive AN as a ‘safe’, familiar option, while on the other hand experiencing AN as a tormenting, dangerous presence.

*It makes me just want to keep hold of the eating disorder, because although I don’t want it, it’s familiar; it’s something I’ve learned; it’s just (p) I don’t know… You think it’s a friend, but it can’t be a friend if it’s tormenting me, like you know, it’s kind of a mixed bag really.* (Shauna)

*There’s Sophie’s world, which is the rational, saying “this is absolutely ridiculous. For goodness sake, you’re dying, you’re X stone X, X stone Y, X stone Z, whatever – you’re dying, that’s ridiculous, don’t be stupid” And the other part is saying “that’s good – keep going – well done”* (Sophie)

### Ambivalence toward recovery

Distinct from participants’ ambivalent feelings toward their eating disorders was ambivalence toward recovery. Participants’ discussion of recovery was filled with mixed feelings; with part of them desperately seeking to overcome AN, and another part of them shying away from the process. One participant described her attitude toward recovery in the context of wanting to find a balance between achieving what health professionals would deem to be a ‘healthy’ weight and being at a dangerously low weight; she indicated that she wishes to hold onto certain valued aspects of AN (i.e. the confidence and positive body image it provides), while letting go of all of its ego-dystonic symptoms:

*I think there’s a medium (weight) in there that no-one discusses…in between those two extremisms, um, but I think – there’s so much I value about my body image etc. and having an eating disorder, but in saying that I don’t want to come across in that I feel a lot more confident when my eating disorder is really strong. What I’m trying to say is that I feel a lot more confident when I feel at an acceptable weight, and that, you know, I’m not in danger.* (April)

Sophie’s mixed feelings toward recovery were highlighted by her wish to either achieve a healthy weight without engaging in the difficult process or have a consequence-free eating disorder:

*It’s like I want a quick fix. I just want to wake up and either be normal and eat normally like everyone else and maintain a healthy weight, or I want to stay at this weight and not be at risk of (p) death (l) basically.* (Sophie)

In addition to participants’ reluctance to let go of some of the more valued aspects of AN, a number of participants expressed ambivalence toward change in the context of feeling powerless toward, or trapped in AN; feeling that AN is an omnipotent force, and that to struggle against it would be associated with more distress than gains:

*(There’s) the box with the eating disorder – where I am – and all of the dreams that I’ve spoken about, you know, all of the fashion, the friendships, the travel, family – everything in that box. There’s this murky bit in the middle and I am now kind of in that murky bit, but I’m grasping so much to try and keep – to get my hand back on the eating disorder box – because at the moment it just feels terrible in the middle…so I just want to crawl back into the familiar even if it is that shit.* (Shauna)

### Values as a beacon of hope

A number of participants used their experiences of AN to highlight the importance of focusing on values as a tool for addressing ambivalence and enhancing motivation to change in AN. The motivating impact of clarifying non-eating disorder related values and goals emerged as a strong theme among this subsection of participants, most of whom, interestingly, presented as being more engaged in the recovery process. Heather highlighted this phenomenon when indicating the value she places on adventure; her desire to re-engage with adventure sports, which was at the time still somewhat impeded by her AN:

*But there are certain other things that I want to do but I can’t do (p) at the moment, but that’s what spurs me on to keep getting better [I: Yeah] because I can’t go backwards.* (Heather)

One participant experienced values-clarification as an important motivational exercise at the very nadir of her eating disorder – a point at which she nearly died, and had to identify reasons for living and fighting against AN:

*I nearly died unfortunately…it’s a shock to really kind of contemplate [I: Mm-hm] what you really value in life, and…what things are worth sticking around for, you know what I mean?* (April)

This experience has convinced her that a focus on personal values is central to the process of recovery from AN:

*What drew me to actually doing (the research) was because I feel that I could relate to a lot to it, and I feel that, um, that a focus on things – on values and beliefs outside your eating disorder – is probably the biggest – the biggest incentive to get you out of – that helps you to stay on the road to recovery.* (April)

When commenting on the themes, however, Sarah suggested that individuals would need to have achieved a certain degree of progress in their recovery before an exercise in values-clarification could become useful:

*Would you not have to already be a bit into recovery for this to be applicable? For this to be an option to try you would first need to be able to identify the values, mine change daily, I feel completely confused about everything.* (Sarah)

Finally, one participant found the process of taking part in the research interview, which focused on personal values, illuminating from a motivational perspective. She identified thinking about her family, and the values she holds in relation to them, as a potential beacon of hope and tool for enhancing motivation to overcome AN:

*…You’ve mentioned so many times, the word family…and that’s something I’m going to go away with. It’s making me – it’s making me actually feel really bad now – um, maybe I didn’t realise just how much (p) it’s affecting them, or (7 s silence) I don’t know how to articulate what I’m trying to say…Maybe that’s a way I – that’s a positive I can take away with me…at the moment that’s really stirred something up inside of me. I’ve got butterflies in my stomach now.* (Sophie)

## Discussion

Considering Domain 1 (‘Balancing Values’), the finding that participants with AN struggle with the notion of balance in personal values is interesting on a number of levels. Participants’ tendency to pursue values such as self-control and discipline in an extreme way calls to mind the controversy surrounding whether characteristics such as perfectionism and rigidity are personality characteristics of people who develop AN or consequences of semi-starvation effects in AN, given how notoriously difficult it is to disentangle AN symptoms from the personality traits of those who develop it [11, 12]. Considering the mixed ability to clarify values among participants in this study, it may be that those who were more focused on recovery had an increased ability to identify values, and thus a stronger sense of meaning and identity outside of their eating disorder.

This study did not provide clarification around whether pursuit of values to an extreme reflects a pre-existing characteristic or semi-starvation effect. Nor did it clarify whether values-clarification can act as a motivating factor for those entrenched in AN, or whether they need to experience a certain degree of progress in recovery (for example a reduction in semi-starvation effects) to make use of this motivational exercise. Regardless of the direction of this relationship, the findings in relation to balancing values and clarification of values have important clinical implications for working with individuals with AN. Specifically, it is important for therapists working with this population to acknowledge and contain the distress, confusion and disempowerment their clients may be experiencing due to feeling compelled to pursue AN-related values (or values in general) in an extreme way. Therapists may find it useful to explore with clients possible ‘semi-starvation’ effects or personality factors in understanding extreme behaviours in pursuit of values, and how these might aid an understanding of feeling ‘stuck’ in AN symptoms [10, 14, 15]. This in turn may support exploration of how to move forward in challenging AN symptoms with a view to pursuing overarching values that are considered incongruent with AN. There may be a role for ACT in addressing the observed difficulties with balance in values, particularly where pliant valuing may be operating [4, 5]. For example, consideration of the fit between individuals’ beliefs and behaviours may help individuals to work toward developing a more sustainable balance in relation to their values, such that AN has less of a compromising effect on their over-arching values [1]. A further clinical implication relates to the potential benefit of spending time supporting individuals to clarify their *true* values, such that they can access tangible and meaningful goals in relation to their recovery and movement beyond AN. An ACT or MET approach could facilitate this process.

The observed mixed fit between AN and personal values supports previous research highlighting the conflict between the ego-syntonic and ego-dystonic symptoms of AN [6]. Participants’ experience of AN as a representation of their true values supports, to a degree, psychological theories of AN, which have highlighted the valued nature of control and thinness, achieved through severe dietary restriction and restraint [16–18].

The finding that control may be valuable due to its role as a coping mechanism or experiential avoidance strategy links with one of the tenets of ACT. This tenet proposes that to hold values because they offer a means of avoiding unwanted internal experiences can be counterproductive, propagating psychological distress [35]. This may represent an important area for intervention among individuals with AN, such that an ACT approach may support individuals to explore and challenge a potential ‘experiential avoidance’ function of control in AN that is ‘dressed up’ as a value. This would enable – in a therapeutic context –consideration of whether some of the valued aspects of AN are having their intended positive effect or rather serving to maintain distress. This could offer a valuable motivation tool.

The perceived ‘misfit’ or incongruence between AN and values may also link with ACT theory, which argues that psychological distress is enhanced through failure to act in line with one’s values [27]. It also fits with a Motivational Interviewing approach to working with ambivalence, which highlights the importance of highlighting discrepancies between people’s behaviour and their overall values and goals [21]. Either way, it supports the argument that psychological interventions should focus on questioning the ‘rightness’ of AN in the context of individuals’ values [1]. Linked to this is the importance of identifying paradoxes in how values are pursued in AN in a therapeutic context. Specifically, it is important to support individuals’ understandings of the ways in which their pursuit of values may in fact be hampered by - as opposed to supported by – their AN symptoms.

It is not surprising, given participants’ experience of a mixed-fit between AN and their values, that significant feelings of ambivalence toward both AN and recovery were observed in this study. Given the finding that ambivalence toward recovery seemed to be more due to a lack of belief in its achievability as opposed to a true lack of desire to move away from AN, it is suggested that clinicians endeavour to redirect discussion away from the term ‘motivation’, which may serve to reinforce a sense of failure or powerlessness in recovery. Discussion around ways in which individuals can be supported to feel more empowered to move more toward behaviours that fit with their values may be more helpful.

Participants’ experiences of using values-clarification as a tool for promoting recovery-orientated behaviour is very encouraging, and indicates that addressing ambivalence in the context of discrepancies between AN and personal values could be a powerful exercise in treatment for AN. However, as mentioned previously, timing may be an issue, as highlighted by Sarah’s belief that a degree of progress in recovery is a prerequisite for this type of work. It is unclear whether there is a stage in recovery from AN when it is most appropriate or, indeed, contra-indicated to focus on values as a vehicle for change. This is an important consideration, given that ACT proponents argue for the use of values as a motivational tool at an early stage of therapy in AN [42]. This study’s findings suggest that individuals with AN have sophisticated insights into the complicated way in which AN interacts with their personal values. Therapists may consider addressing this phenomenon in the context of promoting recovery. In particular, considering participants’ observed feelings of powerlessness against AN, a focus on supporting them to feel (i) empowered in this process, and (ii) that recovery is a realistic or achievable option seems pertinent.

Further in-depth research exploring values in AN is warranted, particularly with regard to the use of values as a motivational tool, and the optimal stage in recovery at which they should be used. Specifically, investigation of whether stage in recovery and/or level of starvation predicts one’s ability to clarify values as a motivational tool would provide useful insights.

### Limitations

When interpreting these results, it is important to recognise the potential impact of a number of factors. Firstly, both inpatients and outpatients with a mixture of diagnoses participated. However, this is not considered to have had a significant impact on findings, given that the main factors that distinguish individuals with AN from those with EDNOS-AN type (based on DSM-IV diagnostic criteria) tend to be physical – a Body Mass Index below 17.5 and amenorrhoea (absence of menstrual periods) – as opposed to psychological. Furthermore, since the publication of DSM-V, participants identified as having EDNOS-AN at the time of recruitment would in fact have met diagnostic criteria for AN. Thus, the psychopathology tends to be similar among these two groups. In fact, two participants in this study had only migrated from a diagnosis of AN to EDNOS-AN a number of weeks prior to participation and would – with the publication of the DSM V following the recruitment and data collection stage – still meet the diagnostic criteria for AN. Also, most participants were receiving some type of psychological therapy at the time of the interview. It is possible that exploring values may have been one element of their therapy and that this may have impacted on participants’ thinking. However, gaining insights from individuals at different stages of recovery added an extra richness to the data, and allowed for exploration of divergences within themes.

## Conclusions

This study provided phenomenological insights into the way individuals with AN experience and relate to their values, in addition to the way in which they perceive their eating disorder to fit with their values. Analysis revealed a difficulty with pursuing values in the extreme and clarifying values. Participants identified AN as having a mixed fit with their values. This was interpreted in the context of existing theory and research relating to ego-syntonic and ego-dystonic symptoms of AN. Finally, exploration of individuals’ experiences of values in relation to recovery – both in terms of ambivalence and motivation – revealed a number of ways in which values may be incorporated into clinical practice and research. What is most exciting for the authors is that this is one of the first studies to focus on personal values in eating disorders, and clearly highlights ways in which therapists can utilise values in the context of enhancing motivation to change, through conversations focused on values clarification or by more explicitly exploring the way in which individuals pursue their values and the fit between AN and values. These conversations, if carried out in a safe, therapeutic context, could act as an aid to therapists in highlighting the discrepancy between clients’ beliefs and behaviour and thus providing motivation to engage in the process of overcoming AN.

## Abbreviations

ACT, Acceptance and Commitment Therapy; AN, Anorexia Nervosa; DSM-IV, Diagnostic and Statistical Manual of Mental Disorders, Fourth Edition; DSM-V, Diagnostic and Statistical Manual of Mental Disorders, Fifth Edition; EDNOS-AN type, Eating Disorder Not Otherwise Specified – Anorexia Nervosa type; EDSs, Eating Disorder Services; IPA, Interpretive Phenomenological Analysis; MET, Motivational Enhancement Therapy; MI, Motivational Interviewing; NHS, National Health Service; P-CAN, Pros and Cons of Anorexia Scale
